# Supplementary material for: Platelet Activation in Ovarian Cancer Ascites: Assessment of GPIIb/IIIa and PF4 in Small Extracellular Vesicles by Nano-Flow Cytometry Analysis
Source: Cancers (Basel). 2022 Aug 24;14(17):4100. doi: 10.3390/cancers14174100 (PMC9454670; doi:10.3390/cancers14174100)
Supplement: Supplementary file 1 [file cancers-14-04100-s001.zip › cancers-1825325-supplementary.pdf]

cancers

MDPI

Supplementary Materials

Platelet Activation in Ovarian Cancer Ascites: Assessment of GPIIb/IIIa and PF4 in Small Extracellular Vesicles by Nano-Flow Cytometry Analysis

Barbara Bortot, Alessandro Mangogna, Ben Peacock Rebecca Lees, Francesco Valle, Marco Brucale, Sara Tassinari, Federico Romano, Giuseppe Ricci and Stefania Biffi *

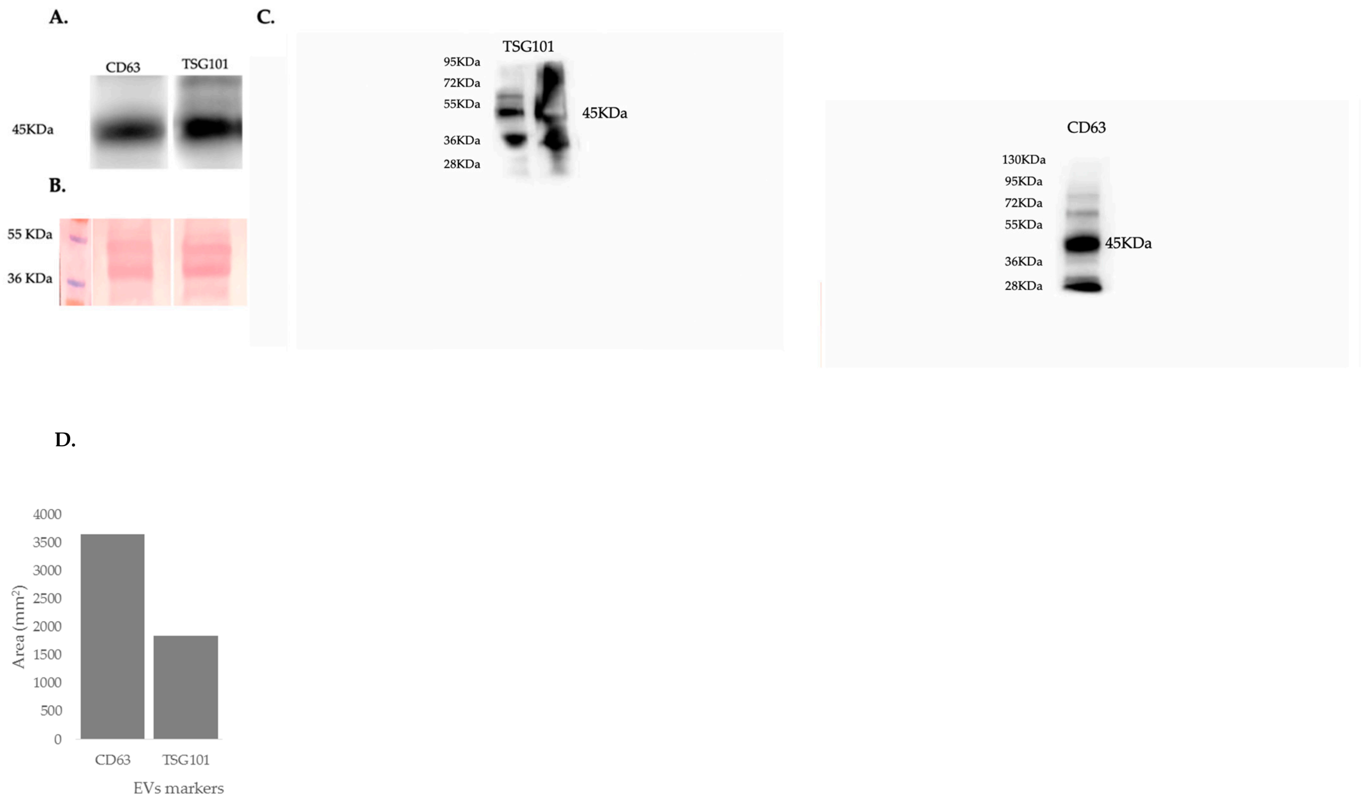

Figure S1. (A) Extracellular vesicle CD63 and TSG101 markers were assayed using Western blotting analysis. (**B**) The Red Ponceau staining shows the transfer of the proteins in the nitrocellulose membrane. (**C**) The uncropped western blots were reported. (**D**) The values of bands area (mm^2) were calculated using the open-source software ImageJ.

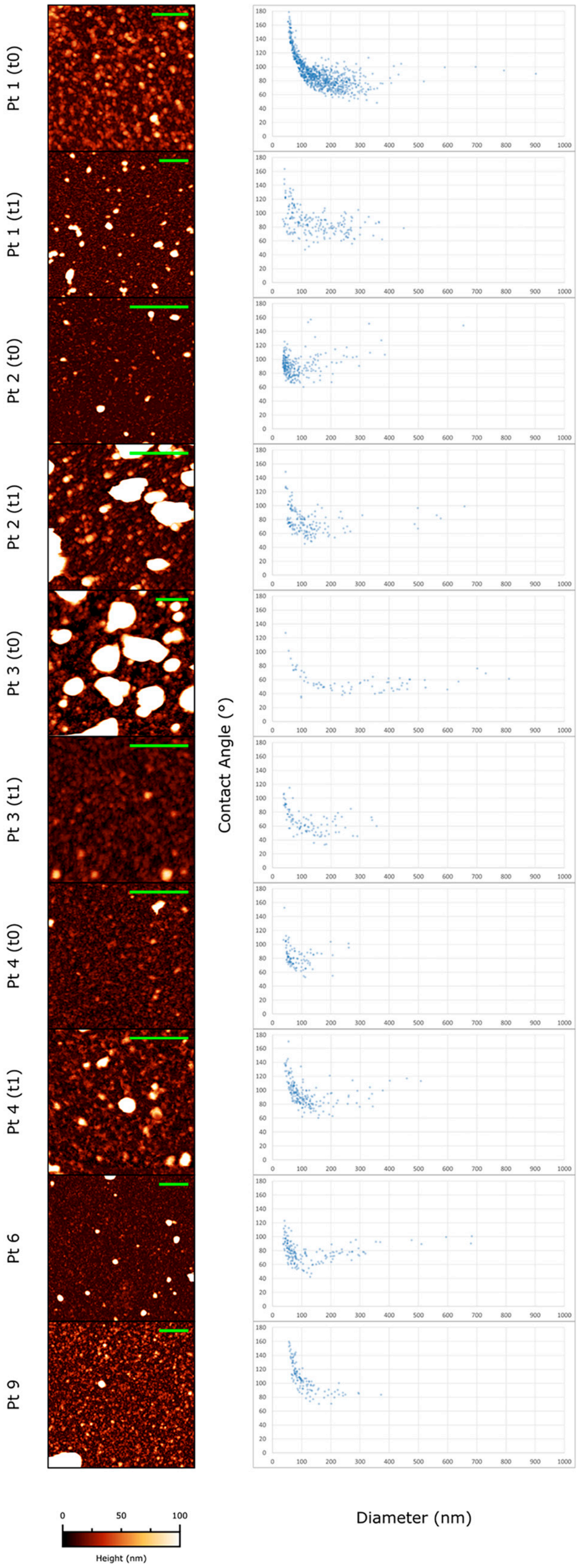

Figure S2. Left column: example AFM micrographs of ten representative samples in the study. All scale bars are 2 μm. Right column: Contact Angle (CA) vs Diameter (D) scatterplots of individual particles measured in each sample via AFM-based single-particle nanomechanical analysis.

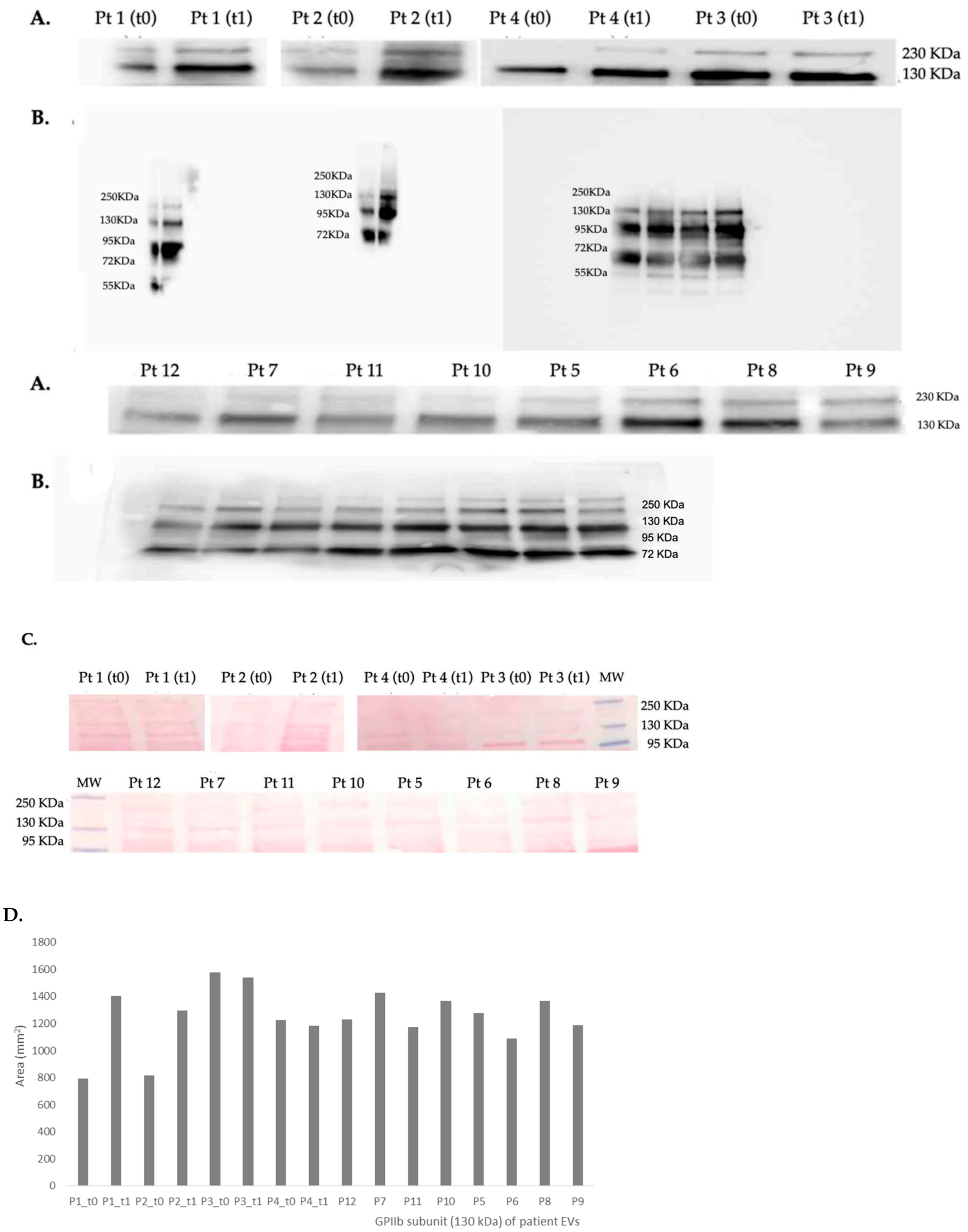

Figure S3. (**A**) Extracellular vesicle GPIIb/IIIa-platelet marker was assayed using Western blotting analysis. (**B)** The corresponding uncropped western blots were reported. (**C)** The Red Ponceau staining shows the transfer of the proteins in the nitrocellulose membrane. (**D)** The values of bands area (mm^2) were calculated using the open-source software ImageJ.
